# A secondary data analysis of a cluster randomized controlled trial: improved cookstoves associated with reduction in incidence of low birthweight in rural Malawi

**DOI:** 10.1093/ije/dyac093

**Published:** 2022-06-02

**Authors:** Rebecca Best, Jullita Malava, Albert Dube, Cynthia Katundu, Fredrick Kalobekamo, Kevin Mortimer, Stephen B Gordon, Moffat Nyirenda, Amelia Crampin, Estelle McLean

**Affiliations:** London School of Hygiene and Tropical Medicine, London, UK; Malawi Epidemiology and Intervention Research Unit, Karonga, Malawi; Malawi Epidemiology and Intervention Research Unit, Karonga, Malawi; Malawi Epidemiology and Intervention Research Unit, Karonga, Malawi; Malawi Epidemiology and Intervention Research Unit, Karonga, Malawi; Liverpool School of Tropical Medicine, Liverpool, UK; Malawi Liverpool Wellcome Trust, Blantyre, Malawi; London School of Hygiene and Tropical Medicine, London, UK; London School of Hygiene and Tropical Medicine, London, UK; Malawi Epidemiology and Intervention Research Unit, Karonga, Malawi; London School of Hygiene and Tropical Medicine, London, UK; Malawi Epidemiology and Intervention Research Unit, Karonga, Malawi

**Keywords:** Maternal health, neonatal health, birth outcomes, birthweight, household pollution, cookstove

## Abstract

**Background:**

In northern rural Malawi, the majority of households cook using open fires and there is also a high burden of adverse birth outcomes. The use of open fires or highly polluting cookstoves is associated with low birthweight in babies. There is mixed evidence on whether implementation of cleaner burning cookstoves reduces the number of babies born with low birthweight.

**Methods:**

This is a secondary analysis of a cluster randomized control trial in Malawi, conducted over 2014–17. Households were randomized to receive improved cookstoves or to continue current practices. For this analysis, the primary outcome was low birthweight in households under routine demographic surveillance, among births occurring within the trial time frame (*N* = 4010). A subset of data with stricter exposure definitions respecting the original randomized allocation was also analysed (*N* = 1050). A causal, forwards modelling approach was used.

**Results:**

The main dataset showed evidence of effect of the intervention on low birthweight [adjusted odds ratio (aOR) 0.69; 95% CI 0.48–0.99, *n* = 2788). The subset analysis lacked power to provide evidence of association between improved cookstoves and low birthweight in the stricter exposure definition (aOR 0.62; 95% CI 0.35–1.09, *n* = 932).

**Conclusions:**

This study provides some evidence that an improved cookstove intervention in rural Malawi reduced the number of babies born with low birthweight by 30%. This direction of the effect was also seen in the subset analysis. The analysis suggests that the intervention reduced the number of infants born prematurely or with intra-uterine growth restriction, indicating that improved cookstoves could be a useful maternal health intervention.

Key Messages

- Around 3 billion people worldwide cook using open fires or inefficient cookstoves, fuelled by biomass, coal or kerosene, accounting for 85% of particulate pollution in low-income countries.
- A growing body of evidence highlights the association with poor birth outcomes and household pollutants, with increases in risk for low birthweight, preterm birth and stillbirth.
- This secondary analysis in northern rural Malawi found evidence that a cleaner-burning, biomass-fuelled cookstove intervention reduced numbers of low birthweight babies born in this population by 31%.
- The intervention included user training, maintenance, two of most efficient stoves available and a solar panel: future programmes need to carefully consider sustainability and implementation.
- Further research on stillbirths, neonatal deaths and other birth outcomes in association with improved cookstoves would aid cost-benefit analysis.

## Introduction

Around 3 billion people worldwide cook using open fires or inefficient cookstoves, fuelled by biomass, coal or kerosene. This accounts for 85% of particulate pollution in low-income countries, causing an estimated 3.8 million premature deaths a year.^1–3^ It is estimated that those who continue to rely on polluting open fires or traditional stoves are exposed to 20 times the recommended World Health Organization (WHO) household target pollutant levels.^1^^,^^2^

Worldwide, women and children are disproportionately exposed to household air pollution, due to traditional gender roles which include a larger volume of cooking and child care for women.^3^ Household pollution health risks for adults and children are well established, and a growing body of evidence highlights the association with poorer birth outcomes: studies have found heavy metals and fine particulate matter in placentas and cord blood, with increases in risk for preterm birth, low birthweight and stillbirth.^4–14^ The pollutants trigger an immune response in the fetus, impact on oxygen concentration capacity, reduce placental health and increase maternal hypertension.^12^^,^^13^ Low birthweight is defined by the WHO as a baby weighing less than 2.5 kg at birth.^16^ These babies are known to be at a higher risk of morbidity and mortality, and are more likely to suffer significant adverse health outcomes across the whole life course.^15–17^

In rural Malawi, gestational age is estimated without use of ultrasound scanning; therefore distinguishing premature birth from intra-uterine growth restriction (IUGR) can be challenging. It is estimated that 30–50% of babies who are born with a low birthweight will have been born prematurely; therefore in this analysis, low birthweight is used as a proxy for both pre-term birth and IUGR.^18^

**Figure 1:**
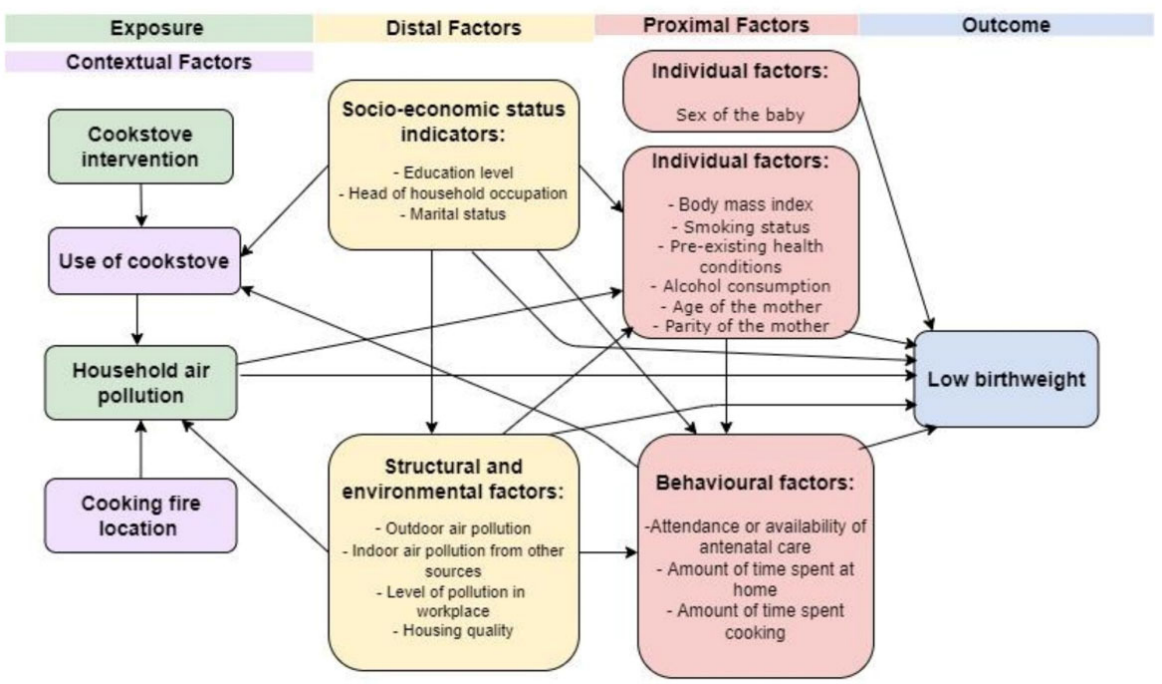
Conceptual framework for the association between a cleaner burning cookstove intervention and poor birth outcomes^1^^,^^3^^,^^7^^,^^8^^,^^19^^,^^20^

Figure 1 presents a conceptual framework illustrating the mechanisms through which improved cookstoves may reduce the number of babies born with low birthweight. As Figure 1 demonstrates, there are confounding variables which are associated with the amount of cookstove emissions and birthweight. These include soci-economic status (SES), marital status and individual factors such as body mass index (BMI).^7^^,^^20^^,^^21^

There is some evidence from observational studies that improved cookstoves reduce low birthweight.^22^^,^^23^ A small cross-sectional study in Nigeria found some evidence that babies born to women using kerosene cookstoves weighed less than those who used cleaner burning cookstoves. This was supported through evidence of an association between a decrease in birthweight and an increase in heavy metals in the cord blood.^24^ Prior to this, in 2002 a larger Guatemalan retrospective study also found reduced birthweight when the women used fires as opposed to cleaner fuel cookstoves.^22^

These findings have not been consistently replicated in the three intervention studies identified.^25–27^ A randomized controlled trial (RCT) in Nigeria in 2017 found evidence that an improved cookstove increased birthweight. A larger RCT in Ghana found no difference in birthweight between an intervention of liquefied petroleum gas or a cleaner-burning biomass-fuelled cookstove with standard cooking practices.^27^ Whole clusters were not randomized, which may have led to contamination of the arms and a dilution of the effect. One further large stepped-wedge RCT in Nepal also found no evidence of an association with a cookstove intervention and birthweight.^25^ However, the reduction in household pollutants was relatively small, remaining well above the WHO recommended levels. This may have been due to an inadequate cookstove or alternative methods also being used.

This analysis aims to partially address the mixed evidence of whether an intervention of cleaner burning cookstoves would improve birthweight, through a secondary analysis of a large, small-area-level, cluster RCT in northern rural Malawi, which randomized clusters of over 4500 households either to a cleaner-burning biomass-fuelled cookstove or to continue standard cooking practices.^28^

## Methods

In northern Malawi, the Karonga Health and Demographic Surveillance Site (HDSS) was established in 2002, within a 150-km² rural area.^28^ This grew to a population of 42 000 people in roughly 8000 households under surveillance by 2016. All births, deaths and in- and out-migrations are captured through a system of ‘key informants’, who meet with fieldworkers on a monthly and annual basis to report events. These events are then followed up at individuals’ houses. Some information about participants (i.e. marital status, education level, occupation and weight and height) and their households (i.e. cooking method and location of cooking fire) have been collected regularly in population-level surveys. Less than 1% of the population in the catchment area decline to participate in the routine surveillance.

A subcluster randomized controlled trial [the Cooking and Pneumonia Study (CAPS)] was conducted in the Karonga HDSS in 2014–17. A total of 8626 households were allocated in geographically defined clusters to two arms of equal size, either to replace their usual cooking practices with two cleaner burning biomass-fuelled cookstoves with solar panels, or to continue cooking practices as normal.^29^ The primary outcome was to compare pneumonia rates in children under 5 years of age (U5) between the arms; therefore eligible households were those with a U5 child in the HDSS. The cookstoves had integrated fans to improve combustion efficiency and user training was provided. In laboratory testing, this model of cookstove was found to be the most efficient model available and reduced smoke by 90%, compared with open fires.^30^

Cookstoves were maintained and replaced when necessary throughout the trial. In a randomly selected 10% of intervention households, monitors of temperature fluctuation were placed in one of the cookstoves as an objective measure of use. The mean number of times the cookstoves were used per day for the monitored cookstoves was 0.51 during the first year and 0.34 in the second year, demonstrating that the cookstoves continued to be used in the intervention arm.^29^ At the end of the trial, the control arm also received the cookstoves. Only 2.6% (226) of households in the HDSS were ineligible, were lost to follow up, did not consent or moved out of the area during CAPS.

Participants in this secondary analysis were women participating in the Karonga HDSS who gave birth in from 2014 to 2018. Their exposure status was assigned according to whether they had a CAPS stove in their household for at least 1 week before the birth. The intervention arm included periods of observation from: (i) households in the CAPS intervention clusters who received a cookstove; and (ii) households in the CAPS control clusters who received a cookstove after completion of the trial from that point forward. The control arm periods of observation were: (i) households that were in CAPS control clusters for the period of the trial; and (ii) non-CAPS households from 2014 onwards. If a woman had more than one birth in the time period, only the first was used unless a subsequent birth had a greater intervention exposure time in pregnancy.

**Figure 2:**
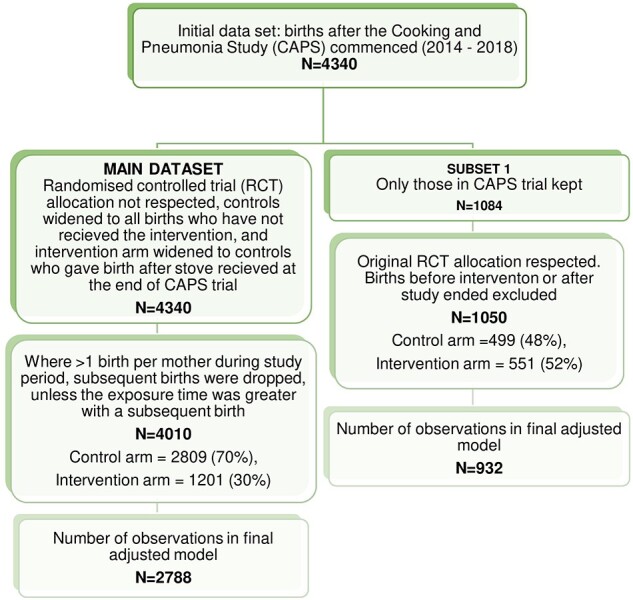
Flowchart of dataset exclusion

Including control households in the intervention group after the trial ended increases the power of the analysis but could have resulted in a dilution of effect if households are less likely to use the cookstove if distributed without the training and support. Therefore, the same analyses were conducted on a smaller subset of the data which only included births in CAPS households during the trial itself, by the initial allocations as presented in Figure 2.

The primary exposure was the cookstove intervention and the primary outcome was low birthweight (defined as weighing under 2.5 kg). When a birth is registered in the HDSS (which may be some time after birth), the midwife-recorded birthweight is transcribed from the patient-held health record of the infant (63.9% of values in the analysis), the mother is asked to report birthweight (21.0% of values) and the weight of the baby is measured by study staff at the time of registration (15.1% of values). No adjustment was made for the day the baby was weighed; however, both the intervention and the control groups had a similar distribution of babies weighed at the time of registration (control: 14.5% and intervention: 16.6%).

At the time of registration, mid upper arm circumference (MUAC) is also measured in the infants. MUAC scores are not used routinely under 6 months of age, but there is evidence that it has a strong correlation with birthweight.^31^ To help understand whether recorded birthweight was informative, despite the discrepancy in measurement methods, a chi square test for association between babies with low birthweight and a low MUAC score was run. There was strong evidence of an association (*P* = <0.001), which suggested that analysing the recorded birthweight would be valuable: 51% of babies with an MUAC score of less than 11 had low birthweight, compared with 25% who did not. Further information on birth (i.e. location and method), the parents’ vital status (if known) and mother’s previous parity is collected during the registration of the birth.

Potential confounders and effect modifiers were assigned from other population surveys in the demographic surveillance area, if the information was gathered within a certain time of the birth: mother’s marital status, household head’s schooling level and occupation reported within 1 year (before or after) of the birth, and cooking fire location within 2 years (before or after) of the birth.

Stata (version 16.0) was used to perform the analysis. Although the data originate from an RCT, only households who had a child under 5 years were eligible for cookstoves and only households with births were included in this analysis. Therefore, as the intervention and control arms might be no longer balanced, confounders were adjusted for as necessary.

A direct approach was used in line with a causal analysis: all confounders identified in the conceptual framework in Figure 1, also available in this dataset, were included in final logistic regression models. For identified variables with >10% missing data, chi square tests of association were carried out between those missing and not missing and key variables. Included confounders were maternal age, parity of the mother, sex of the baby, parents’ education level and marital status. Parents’ education level was selected as a proxy measure for socioeconomic status (SES), as there is good precedent for this as an indicator and head of household occupation contained 26% missing data.^1^ Maternal BMI is known to be associated with birth outcome,^3^ but it was missing in 55% of records. Chi square tests found no association between missing BMI and low birthweight (*P* = 0.58) or trial arm (*P* = 0.7), so multiple imputation was used to allow inclusion of BMI in logistic regression models. As the BMI distribution was skewed, it was converted to the log scale prior to conducting the multiple imputation as this had a more normal distribution; 50 imputations were run, and the imputed BMIs were converted back prior to analysis. Logistic regression models were run with standard errors calculated to reflect that multiple imputation was used.

Conceptually selected potential effect modifiers were tested for interactions. Maternal age, the sex of the baby and parents’ education were identified, as prior research has suggested these variables may determine vulnerability to household pollution: male neonates have been found to be more susceptible to pollutant exposure, younger or older women are more likely to have a low birthweight baby, and those who are at a social or economic disadvantage are more susceptible to negative exposures.^16^^,^^32^ To investigate this, interaction terms were added in the final logistic regression model for the main dataset with the identified variables, obtaining likelihood ratios.

## Results

### Descriptive summary of study population

The sample population consisted of 4340 births between 2014 and 2018. Of these births, 328 (8.3%) had a low birthweight (under 2500 g), 90 (2.1%) were neonatal deaths and 76 (1.8%) were stillbirths.

### Main dataset

The main dataset was made up of 4010 births from different women aged from 12 to 46, over 2014-17: 1201 (30.0%) mothers received the cookstove at least 1 week before birth, with the remaining 2809 women making up the control arm. In the intervention arm, 1041 (86.7%) women received the cookstove in their first trimester of pregnancy, 108 (9.0%) in their second and 52 (4.3%) in their third. In the control arm were 212 (78.2%) low birthweight babies.. The most common parity of birth was 1 or 2 (39.8%), with 84.3% of these births in the control arm. The largest proportion of women were in the age category of 20–24 (29.4%). The sex of the baby was equally distributed. Most births took place in a health facility (98.2%), with nearly all women receiving antenatal care during the pregnancy (99.9%). Table 1 displays the distribution of variables by trial arm.

**Table 1:** Distribution of variables in the study population, by intervention group

	Main dataset, *N *= 4010 (1201, 30.0% in intervention arm)		Subset 1, *N* = 1050 (551, 52.5% in intervention arm)	
	Cookstove intervention control		Cookstove intervention control	
	*N* (%)	*N* (%)	*N* (%)	*N* (%)
**Age of mother (in years)** 0 (0%) missing			**Age of mother (in years)** 0 (0%) missing	
19 and under	139 (11.6)	781 (27.8)	64 (11.6)	46 (9.2)
20-24	312 (26.0)	867 (30.9)	134 (24.3)	158 (31.7)
25-29	321 (26.7)	552 (19.7)	149 (27.0)	142 (28.5)
30-34	244 (20.3)	372 (13.2)	120 (21.7)	99 (19.8)
35-39	148 (12.3)	175 (6.2)	67 (12.2)	42 (8.4)
40 and above	37 (3.1)	62 (2.2)	17 (3.1)	12 (2.4)
Total	1201 (100)	2809 (100)	551 (100)	499 (100)

**Parity of birth** 23 (0.6%) missing			**Parity of birth** 7 (0.7%) missing	
1-2	249 (20.8)	1336 (47.9)	129 (23.6)	138 (27.8)
3-4	510 (42.6)	908 (32.5)	200 (36.6)	200 (40.2)
5-13	437 (36.5)	547 (19.6)	217 (39.7)	159 (32.0)
Total	1196 (100)	2791 (100)	546 (100)	497 (100)

**Sex of baby** 58 (1.5%) missing			**Sex of baby** 17 (1.6%) missing	
Female	595 (50.3)	1379 (49.8)	268 (49.5)	248 (50.3)
Male	587 (49.7)	1391 (50.2)	273 (50.5)	245 (49.7)
Total	1182 (100)	2770 (100)	541 (100)	493 (100)

**Head of household occupation score** ^a^ 825 (20.6%) missing			**Head of household occupation score** 6 (0.6%) missing	
1 Low	40 (4.3)	162 (7.2)	25 (4.6)	35 (7.0)
2 Medium	764 (82.6)	1679 (74.3)	455 (83.2)	393 (79.1)
3 High	121 (13.1)	419 (18.5)	67 (12.3)	69 (13.9)
Total	925 (100)	2260 (100)	547 (100)	497 (100)

**Head of household schooling** 183 (4.6%) missing			**Head of household schooling** 1 (0.1%) missing	
Left, current or never attended primary	383 (32.1)	747 (38.4)	182 (33.0)	138 (27.1
Completed primary	384 (32.2)	740 (28.1)	159 (28.9)	167 (33.5)
Left or current secondary	250 (21.0)	647 (24.6)	124 (22.5)	118 (23.7)
Completed secondary	175 (14.7)	501 (19.0)	86 (15.6)	75 (15.1)
Total	1192 (100)	2635 (100)	551 (100)	498 (100)

**Mother’s marital status** 915 (22.8%) missing			**Mother’s marital status** 12 (1.2%) missing	
Never, ivdorced or widowed	107 (11.8)	373 (17.1)	60 (11.0)	59 (11.94)
Married	802 (88.2)	1813 (82.9)	484 (89.0)	435 (88.1)
Total	909 (100)	2186 (100)	544 (100)	494 (100)

**Mother alive** 59 (1.5%) missing			**Mother alive** 15 (1.5%) missing	
Yes	1182 (100)	2769 (99.9)	541 (100)	493 (100)
Total	1182 (100)	2770 (100)	541 (100)	493 (100)

**Father alive** 103 (2.6%) missing			**Father alive 25** (2.5%) missing	
Yes	1162 (99.8)	2737 (99.8)	533 (99.8)	490 (100)
No	2 (0.2)	6 (0.2)	1 (0.19)	0 (0)
Total	1164 (100)	2743 (100)	534 (100)	490 (100)
**Antenatal care** 58 (1.5%) missing			**Antenatal care** 15 (1.5%) missing	
Yes	1180 (99.8)	2767 (99.9)	540 (99.8)	493 (100)
No	2 (0.2)	3 (0.1)	1 (0.2)	0 (0)
Total	1182 (100)	2770 (100)	541 (100)	493 (100)

**Body mass index** 2208 (55.1%) missing			**Body mass index** 424 (40.1%) missing	
<18.5 kg/m²	49 (6.3)	71 (6.9)	25 (7.5)	16 (5.4)
18.5–24.9 kg/m²	577 (74.3)	756 (73.8)	246 (73.7)	223 (75.6)
>24.9 kg/m²	149 (19.2)	198 (19.3)	63 (18.9)	56 (19.0)
Total	777 (100)	1025 (100)	334 (100)	295 (100)

**Cooking fire location** 1689 (42.1%) missing			**Cooking fire location** 19 (1.8%) missing	
Outdoors	709 (95.7)	1477 (93.5)	520 (95.1)	456 (94.2)
Indoors	32 (4.3)	103 (6.5)	27 (4.9)	28 (5.8)
Total	741 (100)	1580 (100)	547 (100)	484 (100)

**Season fire location asked** 1689 (42.1%) missing			**Season fire location asked** 19 (1.8%) missing	
Rainy season	300 (40.5)	739 (46.8)	191 (34.9)	191 (34.9)
Dry season	441 (59.5)	841 (53.2)	356 (65.1)	293 (60.5)
Total	741 (100)	1580 (100)	547 (100)	484 (100)

**Place of birth** 0 (0.0%) missing			**Place of birth** 0 (0%) missing	
Facility	1175 (97.8)	2763 (98.4)	537 (97.5)	483 (96.8)
Other	26 (2.2)	46 (1.6)	14 (2.5)	16 (3.2)
Total	1201 (100)	2809 (100)	551 (100)	499 (100)

**Low birthweight** 334 (8.3%) missing			**Low birthweight** 105 (9.93%) missing	
Yes	59 (5.3)	212 (8.2)	23 (4.6)	32 (7.1)
No	1045 (94.7)	2360 (91.8)	475 (95.4)	416 (92.9)
Total	1104 (100)	2572 (100)	498 (100)	448 (100)

**Stillbirth** 0 (0%) missing			**Stillbirth** 0 (0%) missing	
Yes	19 (1.6)	39 (1.4)	10 (1.8)	6 (1.2)
No	1182 (98.4)	2770 (98.6)	541 (98.2)	493 (98.8)
Total	1201 (100)	2809 (100)	551 (100)	499 (100)

**Neonatal death** 0 (0%) missing			**Neonatal death** 0 (0%) missing	
Yes	18 (1.5)	47 (1.7)	8 (1.5)	9 (1.8)
No	1183 (98.5)	2762 (98.3)	543 (98.6)	490 (98.2)
Total	1201 (100)	2809 (100)	551 (100)	499 (100)

a Occupation score is coded as 1 being unskilled, irregular work or unemployed, 2 as farming, fishing or a form of skilled work with an unguaranteed wage, and 3 as professionals or those skilled with a regular wage.

Marital status had 23% missing data. There was no evidence of an association between those who had marital status missing and trial arm (*P *=* *0.14) or low birthweight (*P *=* *0.41). Those who had missing marital status were more likely have a parity of 3–4 (*P *=* *0.03). BMI had 55% missing data, with no association between missing BMI and low birthweight (*P *=* *0.58) or trial arm (*P *=* *0.70) found. Those with missing BMI were more likely to be younger (*P *<0.01) and unmarried (*P *<0.01). All other variables had less than 10% missing data.

### Subset 1

Subset 1 (respecting the original CAPS allocation) is made up of 1050 births from different women aged from 14 to 46, between 2014 and 2017. Differing from the main dataset, 551 (52.5%) of the mothers received the cookstove at least 1 week before birth, with 499 women in the control arm. Table 1 displays the spread of variables by cookstove intervention arm: babies born with low birthweight in the control group numbered 32 (58.2%). The largest proportion of women remained in the age category of 20–24 (27.8%), and 88.5% of women were married, but the parity of the birth in this subset was most likely to be 3 or 4 (36.1%).

There were less than 10% missing data in each variable, except 40% in BMI. There was no association between missing BMI and low birthweight (*P *=* *0.29) or trial arm (*P *=* *0.62). Those with a missing BMI were more likely to be younger (*P *<0.01) and unmarried (*P *<0.01),

### Analysis of low birthweight with cookstove intervention

The crude and adjusted logistic regression models are displayed in Table 2. The adjusted odds ratio (aOR), using multiple imputation for BMI, for the main dataset was 0.69 (95% CI 0.48–0.99), finding evidence for an association between low birthweight and the cookstove intervention. For subset 1, the adjusted OR showed a similar direction of effect at 0.62 (95% CI 0.35– 1.09), but with weak evidence found for this association.

**Table 2:** Crude and adjusted odds ratios for low birthweight and cook stove intervention

	Main dataset			Subset 1		
	Odds ratio	95% confidence interval	Number of Observations in model	Odds ratio	95% confidence interval	Number of Observations in model
Crude odds ratio for poor birth outcome and cook stove intervention	0.63	0.47–0.85	3676	0.63	0.36-1.09	946
Adjusted^a^ odds ratio for poor birth outcome and cook stove intervention (complete case analysis excluding BMI)	0.70	0.49–1.01	2788	0.62	0.35–1.09	932
Adjusted^b^ odds ratio for poor birth outcome and cook stove intervention (complete case analysis including BMI)	0.60	0.37–0.97	1315	0.48	0.22-1.09	539
Adjusted^b^ odds ratio for poor birth outcome and cook stove intervention (including imputed BMIs)	0.69	0.48–0.99	2788	0.62	0.35–1.09	932

a Adjusted for maternal age, parity of the mother, sex of the baby, parents’ education level and marital status.

b Adjusted for maternal age, parity of the mother, sex of the baby, parents’ education level, marital status and body mass index (BMI).

There was no evidence shown of effect modification by any pre-specified variable on the association between low birthweight and cookstove intervention in the main dataset: the likelihood ratio tests were *P *=* *0.24 for parent education level (least educated OR 0.49, 95% CI 0.25–0.97; most educated 0.41, 95% CI 0.14–1.19), *P *=* *0.34 for maternal age category (15–19 years old, OR 0.69, 95% CI 0.30–1.60; 40 years and older, OR 1.78, 95% CI 0.11–29.99) and *P *=* *0.6 for sex of the baby (female, OR 0.75, 95% CI 0.47–1.20; male, OR 0.64, 95% CI 0.37–1.12).

## Discussion

We found some evidence that improved cookstoves reduced the odds of a baby being born with a low birthweight by 31%. This is the largest analysis on this topic to date, and was supported by a more restricted analysis of in-trial births: the same direction and similar magnitude of effect were found but there was weak evidence for this finding, likely reflecting a reduction in power due to fewer participants. This finding is consistent with one previous intervention study and all identified observational studies.^10^^,^^22–24^ Two larger intervention studies did not find evidence for this association, but they reported issues with contamination between the study arms and concerns around the cookstoves’ performance.^25^^,^^26^ CAPS measured reducing but continued use of the cookstoves, and implementation included user training, maintenance and the most efficient stove available.^28^^,^^33^

One limitation of this study is birthweight measurement: 21.0% of birthweights were self-reported. Although birthweight was associated with MUAC measurements, the prevalence of low birthweight in Malawi is estimated to be 14.5% and in our sample it was 8.3%.^18^ However, there is no suggestion that the self-reported birthweights were unevenly distributed across the control and intervention arms. The misclassification is therefore not likely to introduce differential misclassification bias even if systematic under- or over-reporting of birthweights occurred, particularly as the birthweight capture was completely independent of the cookstove study. This likely underestimate of low birthweights may have caused a bias towards the null.

Missing data for marital status in the main dataset is unlikely to have caused important bias: no key patterns of missingness or association were identified. Multiple imputation reduced possible bias and confounding due to missing data in BMI in the final models. Parity also remains partially unadjusted across both datasets: there were too few women in their first pregnancy to create a separate stratum, despite primiparity being known to increase the risk of low birthweight. This residual confounding may have biased towards the null, as more first-time mothers were in the control arm (pregnant women were more likely to receive the intervention if they were older and had more children, due to the original eligibility criteria of an under-5 child).

## Conclusion

This analysis found evidence that an improved cookstove intervention reduced the incidence of low birthweight in babies born in rural Malawi, either through a reduction in premature births or in IUGR. The findings highlight the importance of indoor air quality during pregnancy, specifically in low-income settings. Further larger-scale research is required to determine the effects of improved cookstoves on birth outcomes such as stillbirth and neonatal deaths, with longer data collection post-trial to consider the sustainability of this intervention. Understanding at which point in pregnancy the reduced pollutant exposure may be most beneficial could help target interventions.

Qualitative work has shown that households do not view the higher costs of improved cookstoves as affordable or a worthwhile investment, especially when marketed as a health intervention.^30^ Future trials analysing the effect of a quality cookstove intervention on low birthweight and other important birth outcomes should also evaluate the cost-benefit and acceptability of this intervention.

## Ethics approval

Ethics approval for the trial was granted from the Malawi College of Medicine Research Ethics Committee in Malawi (Ref P.11/12/1308), the Liverpool School of Tropical Medicine Research Ethics Committee in the UK (Ref 12.40) and, for the demographic surveillance, from the Malawi National Health Sciences Research Committee (#419/19).

## Data Availability

The data underlying this article were provided by the Malawi Epidemiology and Intervention Research Unit by permission. Data will be shared on request to the corresponding author with permission of the Malawi Epidemiology and Intervention Research Unit.
